# Supplementary material for: Focusing antibody responses to the fusion peptide in rhesus macaques
Source: bioRxiv. 2023 Jun 27:2023.06.26.545779. Preprint. [Version 1] doi: 10.1101/2023.06.26.545779 (PMC10327030; doi:10.1101/2023.06.26.545779)
Supplement: Supplement 1 [file media-1.pdf]

Supplemental Data.

Focusing antibody responses to the fusion peptide in rhesus macaques

Short Title/ Running Title: Fusion Peptide Targeting in NHPs

Christopher A. Cottrell[1,2,#], Payal P. Pratap[1,2,#], Kimberly M. Cirelli[3], Diane G. Carnathan[2,4], Chiamaka A Enemuo[4], Aleksandar Antanasijevic[1,2], Gabriel Ozorowski[1,2], Leigh M. Sewall[1,2], Hongmei Gao[5], Joel D. Allen[6], Bartek Nogal[1,2], Murillo Silva[8], Jinal Bhiman[9], Matthias Pauthner[10], Darrell J. Irvine[2,8], David Montefiori[5], Max Crispin[6], Dennis R. Burton[2,7,10], Guido Silvestri[2,4], Shane Crotty[2,3,11], and Andrew B. Ward[1,2]*

Author Affiliations

[1] Department of Integrative Structural and Computational Biology, The Scripps Research Institute, La Jolla, CA 92037, USA
[2]International AIDS Vaccine Initiative Neutralizing Antibody Center, the Collaboration for AIDS Vaccine Discovery (CAVD) and Scripps Consortium for HIV/AIDS Vaccine Development (CHAVD), The Scripps Research Institute, La Jolla, CA 92037, USA.
[3]La Jolla Institute for Immunology, La Jolla, CA 92037, USA.
[4]Division of Microbiology and Immunology, Yerkes National Primate Research Center, Emory University, Atlanta, GA 30329, USA.
[5]Duke Human Vaccine Institute and Department of Surgery, Duke University Medical Center Durham, NC, USA.
[6]School of Biological Sciences, University of Southampton, Southampton, SO17 1BJ, UK
[7]Department of Immunology and Microbiology, The Scripps Research Institute, La Jolla, California, USA.
[8]Koch Institute for Integrative Cancer Research, Massachusetts Institute of Technology, Cambridge, MA 02139, USA.
[9]Centre for HIV and STI, National Institute for Communicable Diseases of the National Health Laboratory Service, Johannesburg, South Africa.
[10]Center for HIV/AIDS Vaccine Immunology and Immunogen Discovery and IAVI Neutralizing Antibody Center, Department of Immunology and Microbiology, The Scripps Research Institute, La Jolla, California, USA.
[11]Division of Infectious Disease and Global Public Health, Department of Medicine, University of California, San Diego, La Jolla, California, USA
[*]Correspondence: andrew@scripps.edu
[#]Contributed equally

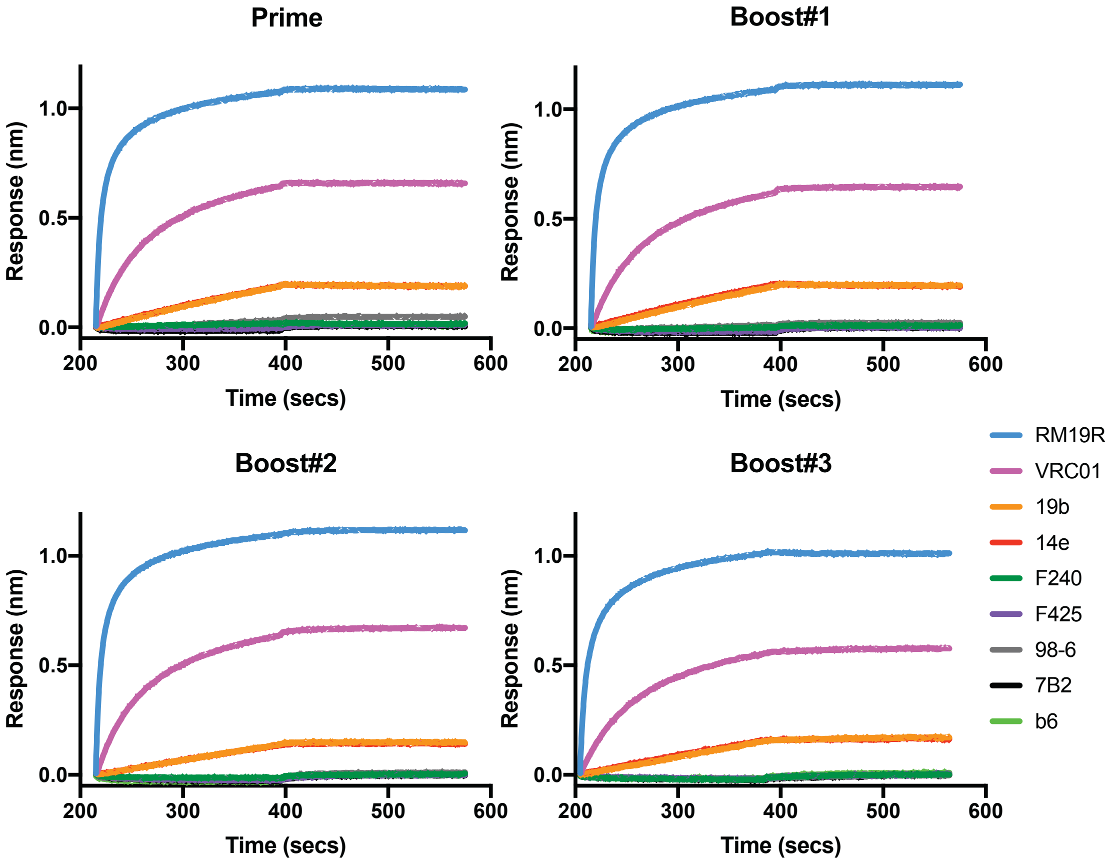

Figure S1. Antigenicity of FP targeting immunogen series. RM19R is a base-binding mAb elicited in a BG505 SOSIP.664 immunized RM (*9*). VRC01 is a CD4 binding site targeting bnAb (*34*) and b6 is a CD4 binding site targeting non-neutralizing mAb. 19b, 14e, and F425 are V3 targeting non-neutralizing mAbs. F240, 7B2, and 98-6 post-fusion gp41 targeting mAbs.

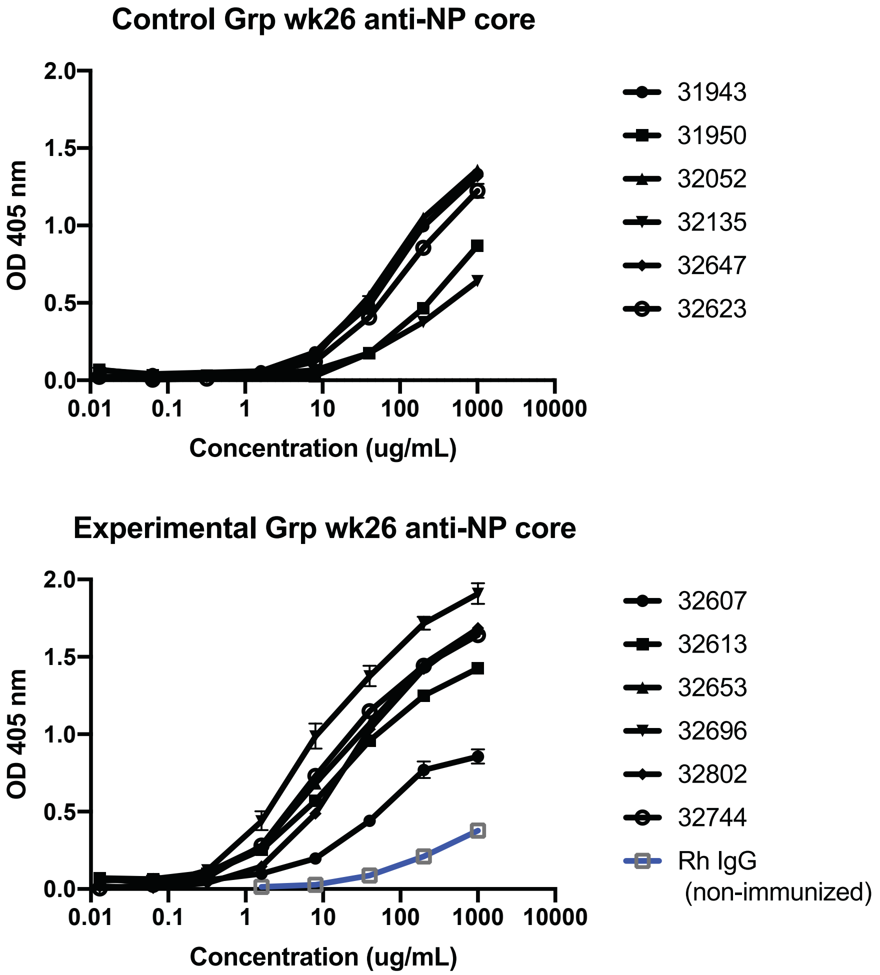

Fig. S2. IgG binding ELISAs for T33-31 nanoparticle core.

IgG purified from week 26 was analyzed at 5-fold dilutions to determine IgG recognition of T33-31 nanoparticle core. Upper panel shows response for the control group monkeys while lower panel shows response for the experimental group monkeys.

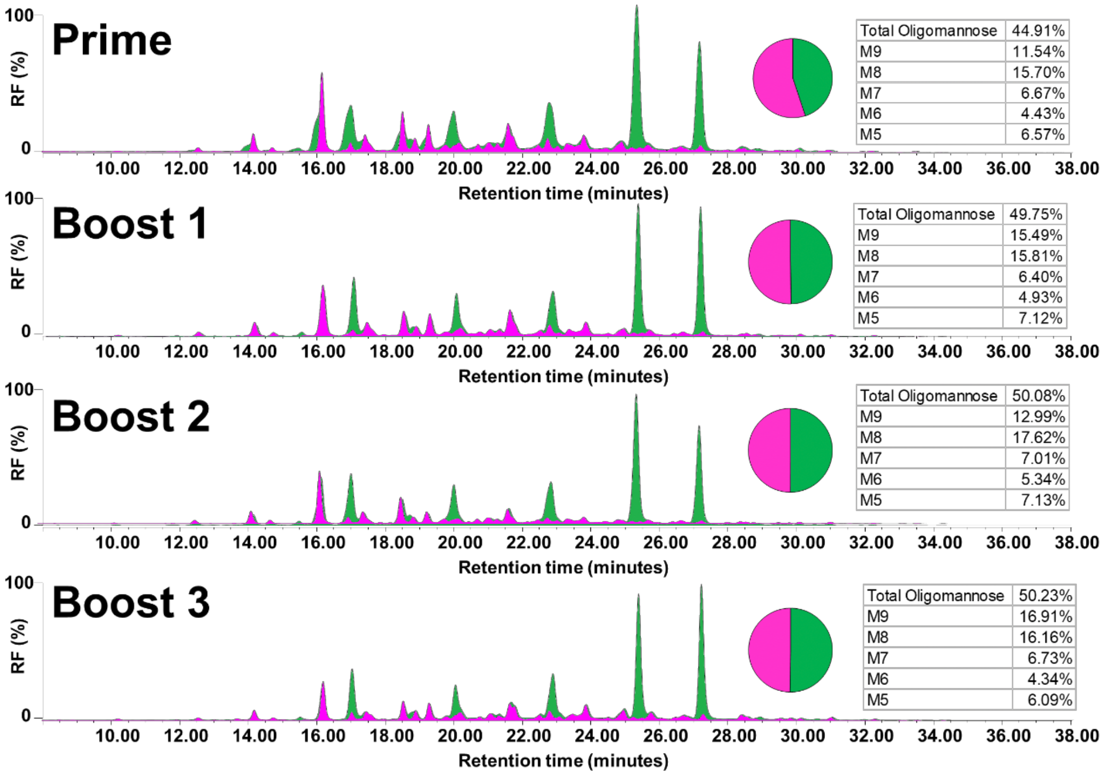

Fig. S3. UPLC analysis of released N-glycans from the sequential immunogens. Procainamide labelled glycans were subjected to endoH digestion that enables the determination and quantification of oligomannose and hybrid-type glycans (green) and complex-type glycans (magenta). Oligomannose-type glycans were quantified using the Waters Empower software.

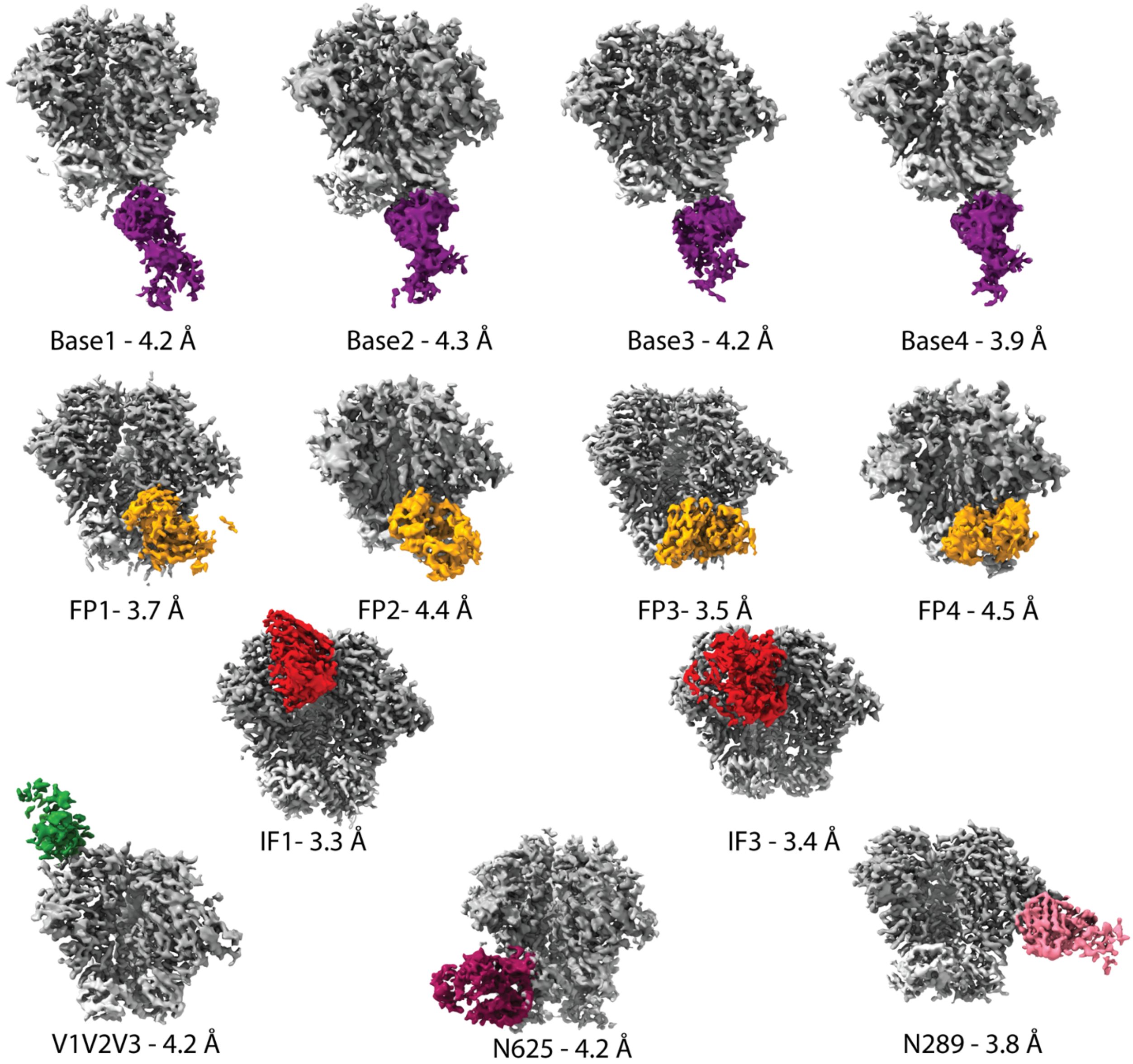

Fig S4. CryoEMPEM High Resolution Maps

Thirteen high resolution (≤4.5 Å) maps were resolved from one polyclonal sample (animal 32613 at week 42 timepoint). Resolved maps include four against the base of the trimer, four maps against the fusion peptide (FP) region, two against the interface (IF) region, one against the V1V2V3 variable region, one against the N625 gp41 glycan and one against N289 glycan of gp120.

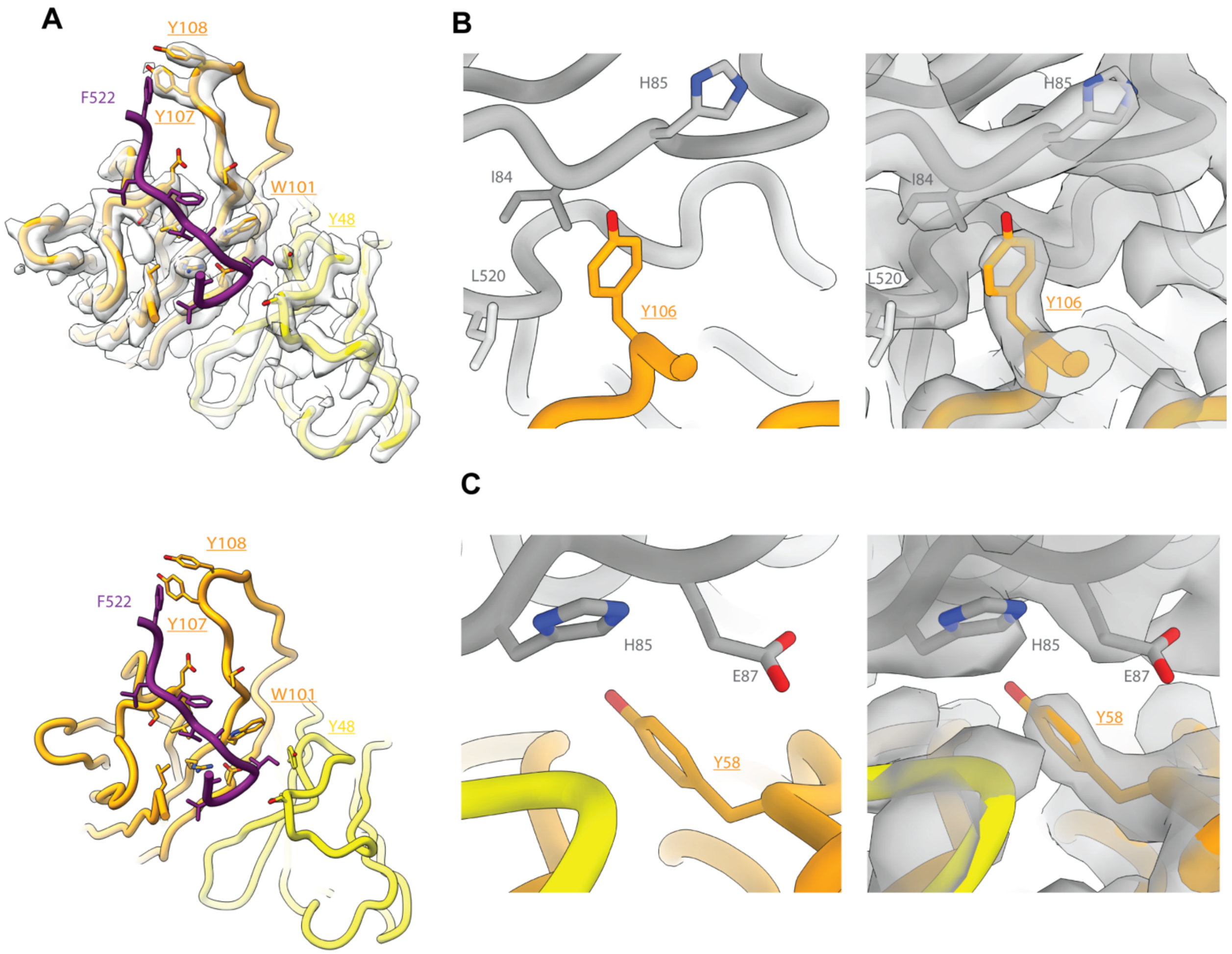

Figure S5. CryoEMPEM Polyclonal Residue Assignments

A) FP3 map and model showing residue predictions (underlined residues) based on density information for FP interaction with HCDR3 and LFW2 aromatic residues. B) FP1 map and model showing residue predictions (underlined residues) based on density information for HCDR3 interaction with C-terminal FP residues and C1 region of gp120. C) FP1 map and model showing residue prediction (underlined residue) based on density information for HCDR2 interaction with C1 region of gp120.

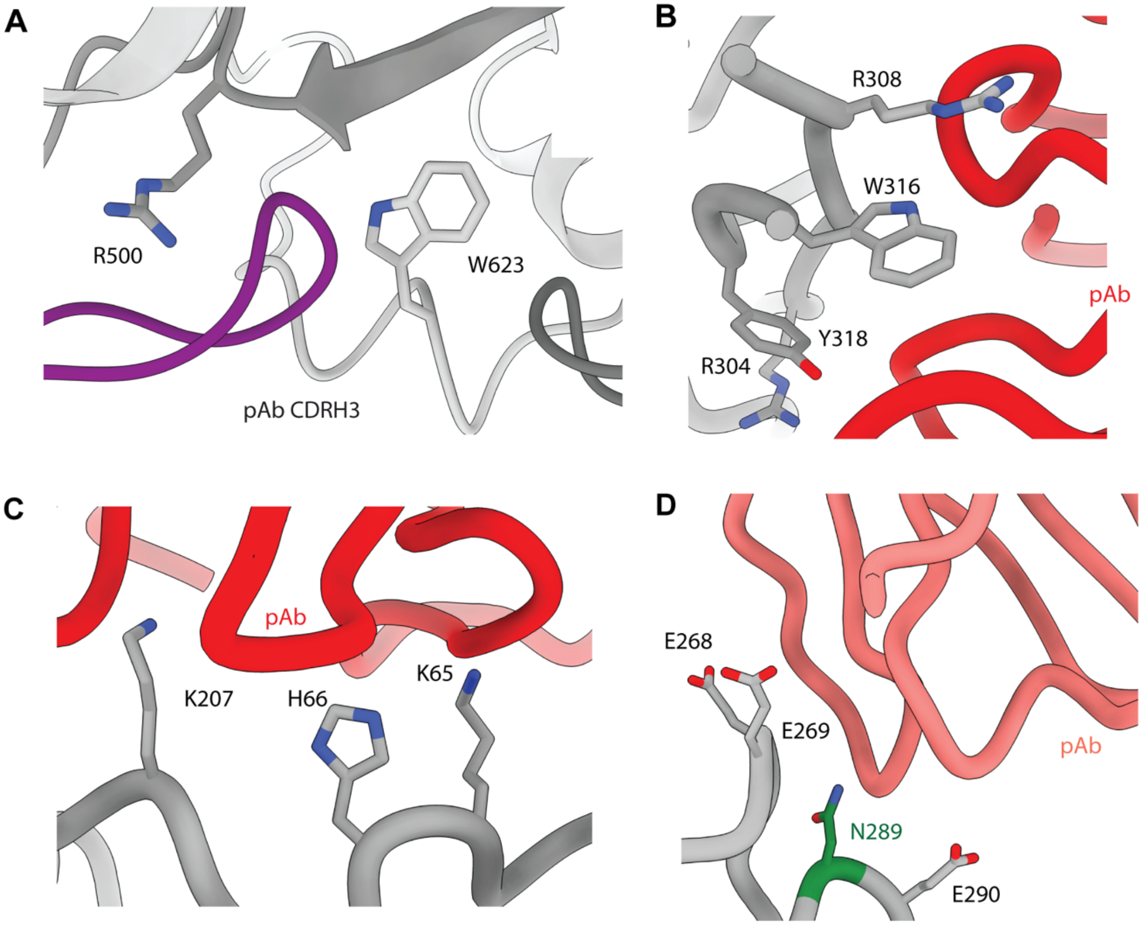

Figure S6. CryoEM Analysis of Off-target Responses

A) Base response elucidated from Base-4 map shows predicted CDRH3 of pAb interaction with base of the trimer. B) IF response elucidated from IF-1 map shows antibody interactions with V3 region of trimer. C) IF response elucidated from IF-3 map shows antibody interactions with C1 region of trimer. D) N289 response shows antibody interaction with C2 region of trimer due to absence of N289 glycan.

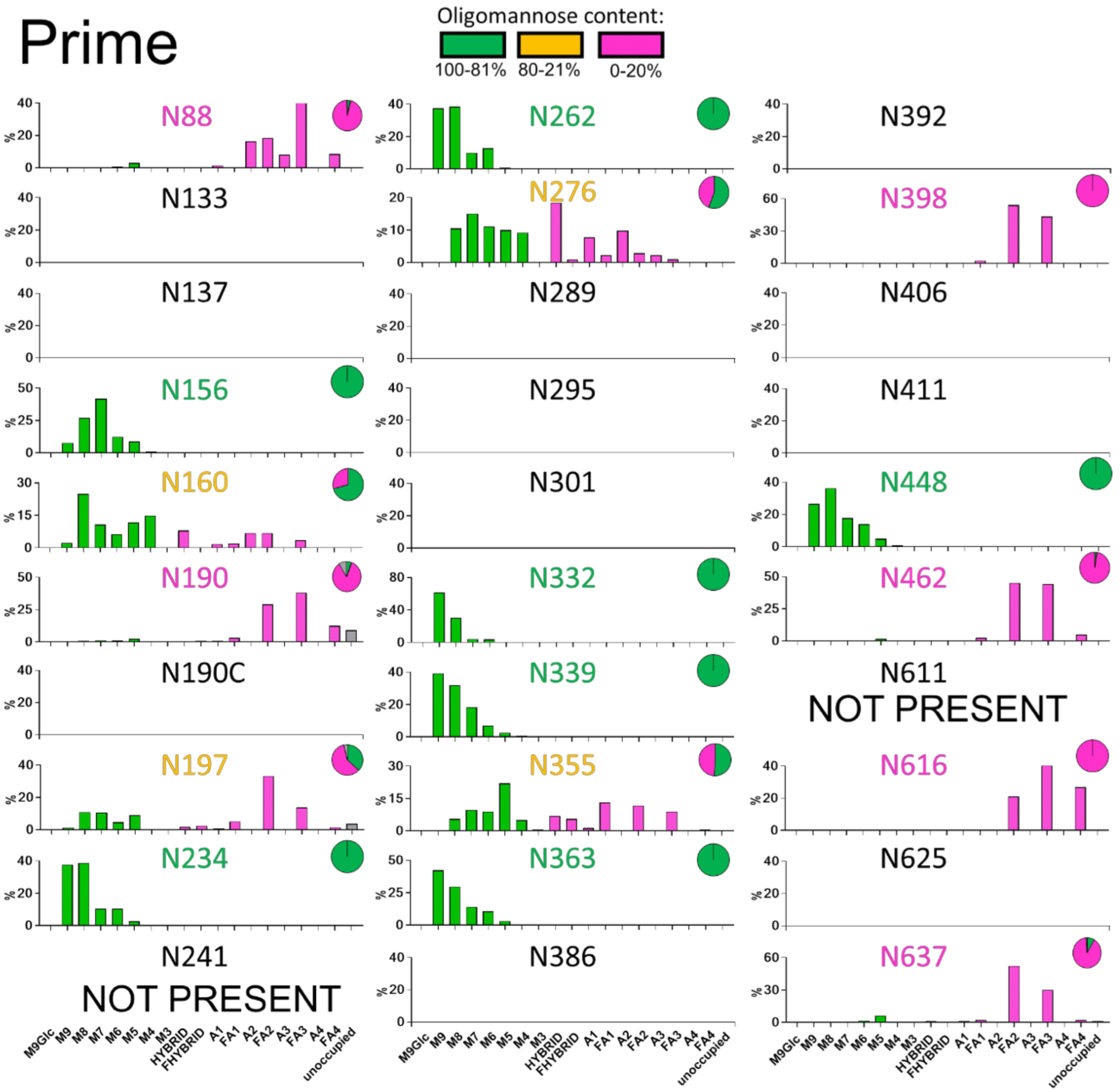

Fig. S7. Site-specific analysis of the Prime immunogen.

The intensity of different glycoforms at a single PNGS were compared and glycopeptides were categorized according to the number of mannose residues (green), hybrids that possess one unprocessed arm and one processed and also the number of processed branches and the presence/absence of fucose for complex-type glycans (magenta). The proportion of unoccupied asparagines at each site is colored grey. Pie charts summing the total oligomannose (M9-M4), complex and unoccupied are shown to the right of each site and the color of the lettering refers to

the % oligomannose at each site, with green letters containing over 80% oligomannose, between 80% and 20% colored orange and less than 20% magenta.

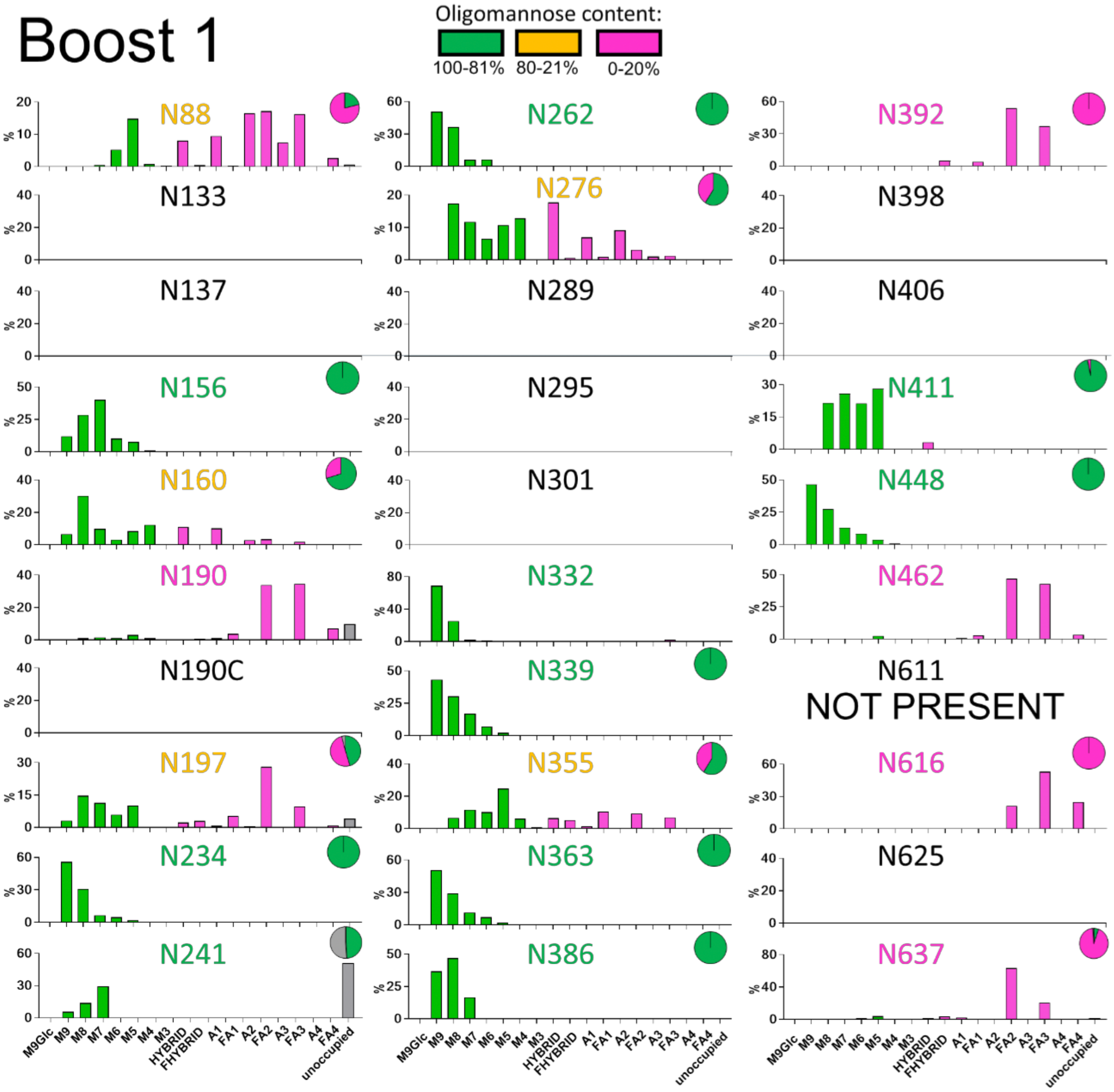

Fig. S8. Site-specific analysis of the Boost#1 immunogen.

Same as in Fig. S7

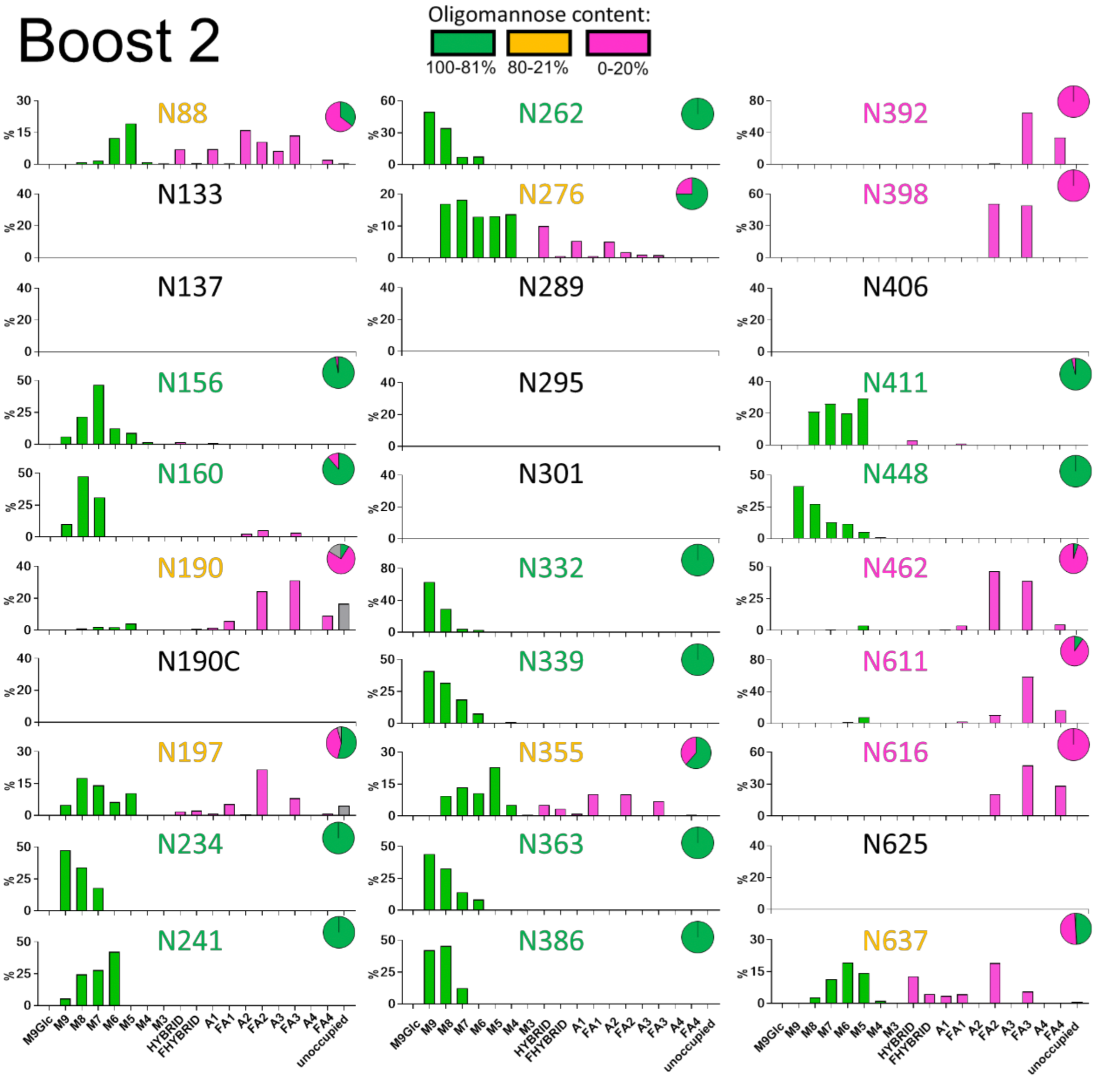

Fig. S9. Site-specific analysis of the Boost#2 immunogen.

Same as in Fig. S7

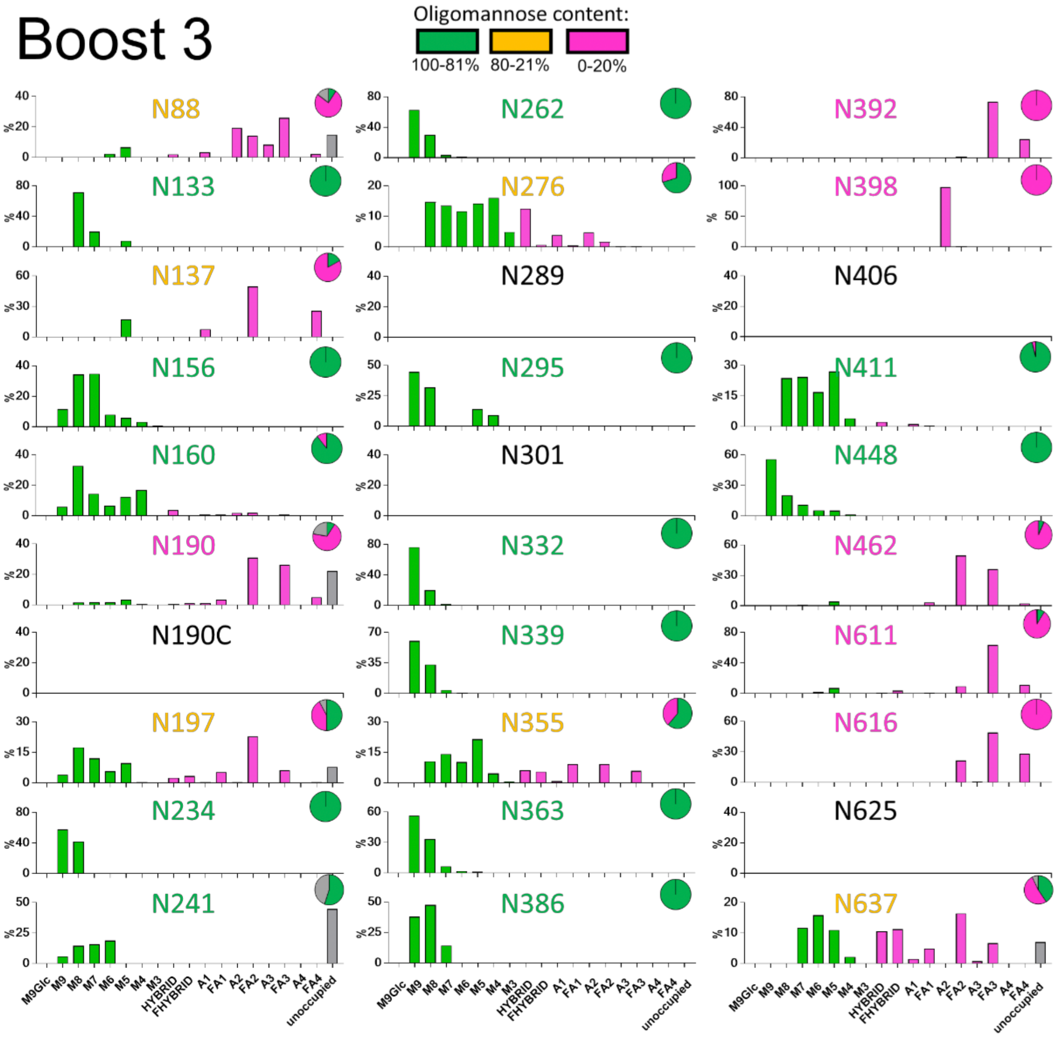

Fig. S10. Site-specific analysis of the Boost#3 immunogen.

Same as in Fig. S7

Table S1. CryoEM data collection, processing and model building statistics.

Map	BG505_BOOST_2 32613 WK 42 Polyclonal Fab Base1	BG505_BOOST_2 32613 WK 42 Polyclonal Fab Base2	BG505_BOOST_2 32613 WK 42 Polyclonal Fab Base3	BG505_BOOST_2 32613 WK 42 Polyclonal Fab Base4
EMDB	EMD-40807	EMD-40808	EMD-40809	EMD-40824
**Data collection**				
Microscope	TFS Titan Krios	TFS Titan Krios	TFS Titan Krios	TFS Titan Krios
Voltage (kV)	300	300	300	300
Detector	Gatan K3	Gatan K3	Gatan K3	Gatan K3
Recording mode	Super-resolution	Super-resolution	Super-resolution	Super-resolution
Nominal magnification (without super-res)	22,500x	22,500x	22,500x	22,500x
Movie micrograph pixelsize (Å; super-res)	0.5155	0.5155	0.5155	0.5155
Number of frames	71	71	71	71
Total dose ($e^-/Å^2$)	40.9	40.9	40.9	40.9
Defocus range (μm)	-2.0 to -0.5	-2.0 to -0.5	-2.0 to -0.5	-2.0 to -0.5
**EM data processing**				
Number of movie micrographs	6015	6015	6015	6015
Number of molecular projection images in map	56597	44865	47856	53702
Symmetry	C1	C1	C1	C1
Map pixel size	1.031	1.031	1.031	1.031
Map resolution (FSC 0.143; Å)	4.2	4.3	4.2	3.9
Map sharpening B-factor ($Å^2$)	-111.11	-130.81	-103.58	-98.19
**Structure building and validation**				
Number of atoms in deposited model				
Non-hydrogen atoms	*NA*	*NA*	*NA*	15170
Protein residues				1866
ligands				67
MolProbity score				1.10
Clashscore				1.27
EMRinger score				2.83
d FSC model (0.5; Å)				4.0
RMSD from ideal				
Bond length (Å)				0.021
Bond angles (˚)				1.678
Ramachandran plot				
Favored (%)				96.26
Allowed (%)				3.41
Outliers (%)				0.33
Side chain rotamer outliers (%)				0.41
Cβ outliers (%)				0.00
PDB				8SWX

Map	BG505_BOOST_2 32613 WK 42 Polyclonal Fab FP1	BG505_BOOST_2 32613 WK 42 Polyclonal Fab FP2	BG505_BOOST_2 32613 WK 42 Polyclonal Fab FP3	BG505_BOOST_2 32613 WK 42 Polyclonal Fab FP4
EMDB	EMD-40810	EMD-40803	EMD-40981	EMD-40804
**Data collection**				
Microscope	TFS Titan Krios	TFS Titan Krios	TFS Titan Krios	TFS Titan Krios
Voltage (kV)	300	300	300	300
Detector	Gatan K3	Gatan K3	Gatan K3	Gatan K3
Recording mode	Super-resolution	Super-resolution	Super-resolution	Super-resolution
Nominal magnification	22,500x	22,500x	22,500x	22,500x
Movie micrograph pixelsize (Å)	0.5155	0.5155	0.5155	0.5155
Number of frames (Falcon 4 EER fractions)	71	71	71	71
Total dose (e^-/Å^2)	40.9	40.9	40.9	40.9
Defocus range (µm)	-2.0 to -0.5	-2.0 to -0.5	-2.0 to -0.5	-2.0 to -0.5
**EM data processing**				
Number of movie micrographs	6015	6015	6015	6015
Number of molecular projection images in map	63946	40085	88235	31418
Symmetry	C1	C1	C1	C1
Map pixel size	1.031	1.031	1.031	1.031
Map resolution (FSC 0.143; Å)	3.7	4.3	3.5	4.4
Map sharpening B-factor (Å^2)	-102.76	-134.84	-96.84	-103.84
**Structure building and validation**				
Model Composition				
Non-hydrogen atoms	15147	*NA*	14900	*NA*
Protein residues	1895		1852	
ligands	59		64	
MolProbity score	0.96		0.71	
Clashscore	0.97		0.64	
EMRinger score	2.95		3.20	
d FSC model (0.5; Å)	3.9		3.6	
RMSD from ideal				
Bond length (Å)	0.022		0.021	
Bond angles (˚)	1.706		1.625	
Ramachandran plot				
Favored (%)	97.03		97.99	
Allowed (%)	2.76		1.96	
Outliers (%)	0.22		0.06	
Side chain rotamer outliers (%)	0.14		0.28	
Cβ outliers (%)	0.00		0.00	
PDB	8SW7		8T2E	

**Map**	**BG505_BOOST_2 32613 WK 42 Polyclonal Fab IF1**	**BG505_BOOST_2 32613 WK 42 Polyclonal Fab IF3**	**BG505_BOOST_2 32613 WK 42 Polyclonal Fab V1V2V3**	**BG505_BOOST_2 32613 WK 42 Polyclonal Fab N625**
EMDB	EMD-40822	EMD-40823	EMD-40805	EMD-40806
**Data collection**				
Microscope	TFS Titan Krios	TFS Titan Krios	TFS Titan Krios	TFS Titan Krios
Voltage (kV)	300	300	300	300
Detector	Gatan K3	Gatan K3	Gatan K3	Gatan K3
Recording mode	Super-resolution	Super-resolution	Super-resolution	Super-resolution
Nominal magnification (without super-res)	22,500x	22,500x	22,500x	22,500x
Movie micrograph pixelsize (Å; super-res)	0.5155	0.5155	0.5155	0.5155
Number of frames	71	71	71	71
Total dose ($e^-/Å^2$)	40.9	40.9	40.9	40.9
Defocus range (μm)	-2.0 to -0.5	-2.0 to -0.5	-2.0 to -0.5	-2.0 to -0.5
**EM data processing**				
Number of movie micrographs	6015	6015	6015	6015
Number of molecular projection images in map	231106	99215	54747	38218
Symmetry	C1	C1	C1	C1
Map pixel size	1.031	1.031	1.031	1.031
Map resolution (FSC 0.143; Å)	3.3	3.4	4.2	4.2
Map sharpening B-factor ($Å^2$)	-103.59	-95.39	-134.42	-113.30
**Structure building and validation**			NA	
Number of atoms in deposited model				
Non-hydrogen atoms	14906	14840		
Protein residues	1853	1881		
ligands	60	43		
MolProbity score	0.93	0.95		
Clashscore	0.81	0.85		
EMRinger score	3.83	3.85		
d FSC model (0.5; Å)	3.4	3.6		
RMSD from ideal				
Bond length (Å)	0.021	0.021		
Bond angles (˚)	1.635	1.679		
Ramachandran plot				
Favored (%)	96.99	96.85		
Allowed (%)	2.89	3.15		
Outliers (%)	0.11	0.00		
Side chain rotamer outliers (%)	0.21	0.00		
Cβ outliers (%)	0.00	0.00		
PDB	8SWV	8SWW		

**Map**	**BG505_BOOST_2 32613 WK 42 Polyclonal Fab N289**
EMDB	EMD-40982
**Data collection**	
Microscope	TFS Titan Krios
Voltage (kV)	300
Detector	Gatan K3
Recording mode	Super-resolution
Nominal magnification (without super-res)	22,500x
Movie micrograph pixelsize (Å; super-res)	0.5155
Number of frames	71
Total dose ($e^-/Å^2$)	40.9
Defocus range (µm)	-2.0 to -0.5
**EM data processing**	
Number of movie micrographs	6015
Number of molecular projection images in map	75994
Symmetry	C1
Map pixel size	1.031
Map resolution (FSC 0.143; Å)	3.8
Map sharpening B-factor ($Å^2$)	-115.80
**Structure building and validation**	
Number of atoms in deposited model	
Non-hydrogen atoms	14097
Protein residues	1762
ligands	56
MolProbity score	0.96
Clashscore	0.72
EMRinger score	2.58
d FSC model (0.5; Å)	4.0
RMSD from ideal	
Bond length (Å)	0.021
Bond angles (˚)	1.678
Ramachandran plot	
Favored (%)	96.52
Allowed (%)	3.42
Outliers (%)	0.06
Side chain rotamer outliers (%)	0.22
Cβ outliers (%)	0.00
PDB	8T2F

Table S2. BG505 pseudovirus neutralization data weeks 24, 26 and 42.

Study Group	Animal ID	Week	SVA-MLV	BG505	BG505/T332N	BG505/T332N.N611A	BG505/T332N.T465N	BG505/T332N.133aN+136aA
Control	31943	24	<20	**2453**	**1409**	**1383**	**621**	**409**
Control	31950	24	<20	**202**	**217**	**323**	**60**	**240**
Control	32052	24	<20	**613**	**866**	**836**	**68**	**714**
Control	32135	24	<20	**318**	**290**	**333**	<20	**347**
Control	32647	24	<20	<20	<20	<20	<20	<20
Control	32623	24	<20	**419**	**584**	**804**	**75**	**580**
Experimental	32607	24	<20	**356**	**200**	**398**	**50**	**57**
Experimental	32613	24	<20	<20	<20	**282**	<20	<20
Experimental	32653	24	<20	22	22	**227**	<20	28
Experimental	32696	24	<20	24	**74**	**558**	**46**	24
Experimental	32802	24	<20	<20	22	**676**	<20	<20
Experimental	32744	24	<20	23	<20	**174**	<20	<20
Control	31943	26	<20	**2069**	**1098**	**1000**	**643**	**199**
Control	31950	26	<20	**104**	**134**	**159**	**50**	**137**
Control	32052	26	<20	**305**	**314**	**412**	28	**365**
Control	32135	26	<20	**197**	**174**	**210**	<20	**231**
Control	32647	26	<20	<20	28	<20	<20	<20
Control	32623	26	<20	**273**	**288**	**527**	**57**	**325**
Experimental	32607	26	<20	**486**	**312**	**483**	**125**	**77**
Experimental	32613	26	<20	<20	21	**256**	<20	<20
Experimental	32653	26	<20	39	33	**183**	21	**50**
Experimental	32696	26	<20	**40**	**91**	**468**	**47**	**50**
Experimental	32802	26	<20	<20	22	**465**	<20	<20
Experimental	32744	26	<20	<20	<20	**94**	<20	22
Control	31943	42	<20	**1,663**	**1,134**	**1,209**	**394**	**393**
Control	31950	42	<20	**1,179**	**1,199**	**1,269**	**397**	**1,190**
Control	32052	42	<20	**315**	**302**	**281**	<20	**322**
Control	32135	42	<20	**4,286**	**4,366**	**5,063**	<20	**5,070**
Control	32647	42	<20	**49**	**88**	73	<20	**74**
Control	32623	42	<20	**1,743**	**1,737**	**1,970**	**521**	**1,777**
Experimental	32607	42	<20	**1,823**	**1,361**	**2,288**	**314**	**638**
Experimental	32613	42	<20	**81**	**68**	**3,589**	**47**	**50**
Experimental	32653	42	<20	**125**	**80**	**534**	<20	<20
Experimental	32696	42	<20	**224**	**234**	**643**	**42**	**37**
Experimental	32802	42	<20	**101**	**98**	**4,356**	**48**	**82**
Experimental	32744	42	<20	**78**	**81**	**475**	**44**	**70**

Values are the serum dilution at which relative luminescence units (RLUs) were reduced 50% compared to virus control wells (no test sample)

Table S3. FP-sensitive pseudovirus panel neutralization data weeks 24, 26, and 42.

Study Group#	Animal ID	Week	25710-2.43	3988.25	0077.V1.C16	CNE19	CNE56	KER2008 .vrc12	Q23.17	286_36	BL01.DG
Control	31943	24	<20	<20	<20	**214**	<20	<20	<20	<20	<20
Control	31950	24	<20	26	<20	24	<20	<20	<20	<20	<20
Control	32052	24	<20	26	<20	<20	<20	<20	<20	<20	<20
Control	32135	24	<20	32	<20	34	<20	<20	<20	24	<20
Control	32647	24	<20	31	<20	<20	<20	<20	<20	<20	<20
Control	32623	24	<20	<20	<20	<20	<20	<20	<20	<20	<20
Experimental	32607	24	<20	<20	<20	<20	<20	<20	<20	<20	<20
Experimental	32613	24	<20	<20	<20	32	<20	<20	<20	<20	<20
Experimental	32653	24	<20	34	<20	<20	<20	<20	<20	<20	<20
Experimental	32696	24	<20	24	<20	20	<20	<20	<20	<20	<20
Experimental	32802	24	<20	<20	<20	<20	<20	<20	<20	<20	<20
Experimental	32744	24	<20	26	<20	<20	<20	<20	<20	<20	<20
Control	31943	26	<20	<20	<20	**136**	<20	<20	<20	<20	<20
Control	31950	26	<20	25	<20	27	<20	<20	<20	<20	<20
Control	32052	26	<20	<20	<20	<20	<20	<20	<20	<20	<20
Control	32135	26	<20	31	<20	21	<20	<20	<20	21	<20
Control	32647	26	<20	**55**	<20	22	<20	<20	<20	<20	36
Control	32623	26	<20	<20	<20	<20	<20	<20	<20	<20	<20
Experimental	32607	26	<20	22	<20	<20	<20	<20	<20	<20	<20
Experimental	32613	26	<20	21	<20	26	<20	<20	<20	<20	<20
Experimental	32653	26	<20	**42**	<20	32	<20	<20	21	23	<20
Experimental	32696	26	22	39	<20	30	<20	<20	27	<20	**41**
Experimental	32802	26	<20	<20	<20	<20	<20	<20	<20	<20	<20
Experimental	32744	26	<20	29	<20	22	<20	<20	<20	<20	<20
Control	31943	42	<20	<20	<20	<20	<20	<20	<20	<20	<20
Control	31950	42	<20	<20	<20	<20	<20	<20	<20	<20	<20
Control	32052	42	<20	<20	<20	<20	<20	<20	<20	<20	<20
Control	32135	42	<20	<20	<20	<20	<20	<20	<20	<20	<20
Control	32647	42	<20	<20	<20	<20	<20	<20	<20	<20	<20
Control	32623	42	<20	<20	<20	<20	<20	<20	<20	<20	<20
Experimental	32607	42	<20	<20	<20	<20	<20	<20	<20	<20	<20
Experimental	32613	42	**53**	**42**	<20	**148**	<20	<20	<20	<20	<20
Experimental	32653	42	**<20**	**27**	<20	**<20**	<20	<20	<20	<20	<20
Experimental	32696	42	<20	<20	nd	<20	nd	<20	<20	nd	<20
Experimental	32802	42	<20	<20	<20	**34**	<20	<20	<20	<20	<20
Experimental	32744	42	**30**	**32**	<20	**37**	<20	<20	<20	<20	<20

Values are the serum dilution at which relative luminescence units (RLUs) were reduced 50% compared to virus control wells (no test sample)

Table S4. Global panel pseudovirus neutralization data.

			ID50 (dilution) in TZM-bl cells									
Study Group#	Animal ID	Week	CEO217	CE1176	25710	CH119	X2278	TRO11	BJOX2000	CNE55	X1632	246F3
Crtl	31943	42	<10	<10	<10	<10	<10	<10	<10	<10	<10	<10
Crtl	31950	42	<10	<10	<10	<10	<10	<10	<10	<10	<10	<10
Crtl	32052	42	<10	<10	<10	<10	<10	<10	<10	<10	<10	<10
Crtl	32135	42	<10	<10	<10	<10	<10	<10	<10	<10	<10	<10
Crtl	32647	42	<10	<10	<10	13	<10	<10	<10	<10	<10	<10
Crtl	32623	42	<10	<10	<10	<10	<10	<10	<10	<10	<10	<10
Expt	32607	42	<10	<10	59	19	<10	<10	<10	<10	<10	<10
Expt	32613	42	<10	13	13	<10	<10	<10	<10	<10	<10	<10
Expt	32653	42	<10	<10	<10	<10	<10	<10	<10	<10	<10	<10
Expt	32696	42	<10	<10	<10	<10	<10	<10	<10	<10	<10	<10
Expt	32802	42	<10	<10	15	<10	<10	<10	<10	<10	12	<10
Expt	32744	42	<10	13	75	<10	<10	<10	<10	<10	15	<10

Values are the serum dilution at which relative luminescence units (RLUs) were reduced 50% compared to virus control wells (no test sample)

Table S5. Immunogen mutations relative to BG505 SOSIP.v5.2

Immunogen	Mutations relative to BG505 SOSIP.v5.2
Prime	M271I-F288L-T290E-P291S-N611Q
Boost#1	P240T-S241N-M271I-F288L-T290E-P291S-N611Q
Boost#2	P240T-S241N-M271I-F288L-T290E-P291S-S613T
Boost#3	H85V-K229N-P240T-S241N-M271I-F288L-T290E-P291S-S613T

Table S6. FACS antibody panel.

Marker	Clone	Flurophore
Viability	n/a	APCe780
CD20	2H7	Ax488
CD4	OKT-4	APCe780
CD8a	RPA-T8	APCe780
CD16	ebioCB16	APCe780
IgG	G18-145	AF700
IgM	G20-127	PerCP-Cy5.5
CD71	L01.1	PE-CF594
CD38	OKT	PE
